# Regulatory Role of the RUNX2 Transcription Factor in Lung Cancer Apoptosis

**DOI:** 10.1155/2022/5198203

**Published:** 2022-12-03

**Authors:** Camila Bernal, Andrea Otalora, Alejandra Cañas, Alfonso Barreto, Karol Prieto, Martin Montecino, Adriana Rojas

**Affiliations:** ^1^Institute of Human Genetics, School of Medicine, Pontificia Universidad Javeriana, 110231 Bogota, Colombia; ^2^Internal Medicine Department, School of Medicine, Pontificia Universidad Javeriana, 110231 Bogota, Colombia; ^3^Internal Medicine Department, Hospital Universitario San Ignacio, 110231 Bogota, Colombia; ^4^Immunology and Cellular Biology Research Group, Microbiology Department, Pontificia Universidad Javeriana, 110231 Bogota, Colombia; ^5^Institute of Biomedical Sciences, School of Medicine and School of Life Sciences, Universidad Andres Bello, 8370035 Santiago, Chile

## Abstract

Lung cancer is the leading cause of cancer death globally. Numerous factors intervene in the onset and progression of lung tumors, among which the participation of lineage-specific transcription factors stands out. Several transcription factors important in embryonic development are abnormally expressed in adult tissues and thus participate in the activation of signaling pathways related to the acquisition of the tumor phenotype. RUNX2 is the transcription factor responsible for osteogenic differentiation in mammals. Current studies have confirmed that RUNX2 is closely related to the proliferation, invasion, and bone metastasis of multiple cancer types, such as osteosarcoma, breast cancer (BC), prostate cancer, gastric cancer, colorectal cancer, and lung cancer. Thus, the present study is aimed at evaluating the role of the RUNX2 transcription factor in inhibiting the apoptosis process. Loss-of-function assays using sh-RNA from lentiviral particles and coupled with Annexin/propidium iodide (PI) assays (flow cytometry), immunofluorescence, and quantitative PCR analysis of genes related to cell apoptosis (*BAD*, *BAX*, *BCL2*, *BCL-XL*, and *MCL1*) were performed. Silencing assays and Annexin/PI assays demonstrated that when RUNX2 was absent, the percentage of dead cells increased, and the expression levels of the *BCL2*, *BCL-XL*, and *MCL1* genes were downregulated. Furthermore, to confirm whether the regulatory role of RUNX2 in the expression of these genes is related to its binding to the promoter region, we performed chromatin immunoprecipitation (ChIP) assays. Here, we report that overexpression of the *RUNX2* gene in lung cancer may be related to the inhibition of the intrinsic apoptosis pathway, specifically, through direct transcriptional regulation of the antiapoptotic gene *BCL2* and indirect regulation of *BCL-XL* and *MCL1.*

## 1. Introduction

In 2020, lung cancer (LC) represented about 11.4% of new cases among all types of cancer, with 18% of all deaths caused by cancer globally [1]. Lung cancer is classified into two broad categories: non-small-cell lung cancer (NSCLC) and small cell lung cancer (SCLC). NSCLC is the most frequent worldwide and is divided into adenocarcinoma (ADC), squamous cell carcinoma (SCC), and large cell carcinoma, with incidences of 40%, 25-30%, and 5-10%, respectively [2]. Despite the high frequency of this cancer type, patients are currently diagnosed in advanced stages of the disease, when the effectiveness of most of the existing treatments is lost, causing life expectancy to be no more than five years after diagnosis [3]. Understanding the cellular and molecular biology of lung cancer facilitates the search for early biomarkers that can be used for diagnosis or as therapeutic targets.

Cancer is a disease characterized by the uncontrolled division of cells called neoplastic cells [4]. These cells originate locally in a primary tissue and can spread through blood and lymphatic systems, invading any other type of tissue or organ [4]. Eventually, for normal cells to be converted to a neoplastic state, specific capacities must be acquired as described by Hanahan and Weinberg in [5]. In this way, it has been described that certain critical transcription factors necessary for embryonic development may exhibit aberrant expression in adult tissue during the initiation and progression of a neoplasm [6]. RUNX2 is a critical transcription factor for osteogenic differentiation in mammals. Its aberrant expression has been directly related to favoring epithelial-mesenchymal transition (EMT) in lung cancer and avoiding apoptotic processes in breast and thyroid cancer [7–10].

Resistance to cell death is one of the hallmarks of cancer and is characterized by interruption and deregulation of the mechanisms that drive the apoptotic process. The effector activity of apoptosis is orchestrated by members of the protease family called caspases, whose activation can occur in two ways: the extrinsic and intrinsic pathways. The intrinsic pathway, also known as the mitochondrial apoptosis pathway, involves a variety of stimuli that act on multiple targets within the cell and are mitochondrial-initiated events [11]. The control and regulation of these mitochondrial apoptotic events occur through Bcl-2 family proteins [12], and their deregulation is related to tumorigenesis and cancer cell resistance to anticancer treatments [12].

In this study, we found that the increased expression of RUNX2 in the A549 cell line is related to the evasion of apoptosis in lung cancer. This was demonstrated by RUNX2 loss-of-function assays linked to Annexin/PI assays, immunofluorescence, mRNA expression analysis, and ChIP, where it was shown that RUNX2 inhibits the intrinsic apoptosis pathway through transcriptional regulation of the antiapoptosis genes *BCL2*, *BCL-XL*, and *MCL1*, which showed significantly decreased gene expression under the *sh-RUNX2* knockdown condition. These molecular changes affected the survival of A549 lung cancer cells.

## 2. Materials and Methods

### 2.1. Cell Culture

The A549 human alveolar basal epithelial cell adenocarcinoma cell line (ATCC® CCL-185™) was cultured in Dulbecco's modified Eagle's medium (DMEM) supplemented with 10% fetal bovine serum (FBS) and 5% antibiotics (ampicillin and streptomycin). All cell cultures were incubated at 37°C in 5% CO_2_ with a relative humidity of 95%. Cells were harvested upon reaching 80-90% confluence. For the experiments evaluating cell death due to apoptosis, the drug doxorubicin (DOXO) (0.2 *μ*m) was used as an inducing agent. Mycoplasma detection test was performed in the A549 cell line.

This study included tissue specimens from one patient with primary NSCLC with histopathologically verified lung mass. The patient underwent surgical resection at the Hospital Universitario San Ignacio, Bogota. The solid tumor was mechanically disaggregated under a stereoscope. Cells were cultured in base C growth medium supplemented with 5% fetal bovine serum [13].

This research was performed under the Colombian Ministry of Health Guidelines (008430-1993) and approved by the Pontificia Universidad Javeriana, School of Medicine Ethics Committee. After a written and signed informed consent form was obtained from the patient, all procedures were conducted.

### 2.2. Production and Lentiviral Infection of A549 Cells

Gene silencing assays were performed using sh-RNA (short-hairpin-RNA) in the A549 cell line through lentiviral infection using a three-plasmid system. The HEK293T cell line (Life Technologies) was cultured in 60 mm plates in DMEM supplemented with 10% SFB. Lipofectamine 2000 (Invitrogen) and the plasmids: pCMV-VSV-G (0.2 *μ*g/*μ*l), pCMV-dR8.91 (*Δ*89) (0.512 *μ*g/*μ*l), and pLKO.1-sh-RNA (3.3 *μ*g/*μ*l) at a ratio of 1 : 2 : 3 were used for transfection following the manufacturer's instructions, with a maximum total DNA of 10 mg per plate. pLKO.1 EV sh-Ctrl was used as a control (4.275 *μ*g/*μ*l). At 24 hours posttransfection, the culture medium was changed to DMEM supplemented with 1% FBS, and after 72 hours, the supernatant containing the viral particles was collected. This supernatant was filtered through a PVDF filter with 0.22 *μ*m pores. Transduction of A549 cells was conducted in six-well plates in which 150.000 cells were seeded with 2 ml of DMEM supplemented with 10% SFB to 70% confluence, followed by the use of Polybrene (Sigma-Aldrich) with a volume of 8 *μ*l per plate.

### 2.3. Nuclear Extracts and Western Blotting

All Western blotting procedures were conducted with nuclear extracts of the A549 cell line obtained 48 hours after transfection with *sh-RUNX2* or *sh-Ctrl*. The nuclear extracts were extracted using the Dingman method. The total nuclear extract proteins were quantified using the Bradford technique. Then, 25 *μ*g of protein was separated via SDS-PAGE. Subsequently, the proteins were transferred to a nitrocellulose membrane, and the nonspecific binding sites were block by incubation of the membrane in 5% milk solution in TBS-Tween for one hour, followed by an “overnight” incubation at 4°C with the primary antibodies anti-RUNX2 (NBP2-24755SS, Novus Biologicals) and antihistone H3 (ab1791, Abcam).

### 2.4. Reverse Transcriptase and Quantitative Real-Time PCR (RT-qPCR)

Total RNA extraction was performed with TRIzol (Ambion Life Technologies) according to the manufacturer's specifications. Briefly, 2 *μ*g of the total RNA was quantified using NanoDrop™ 2000c spectrophotometer (Thermo Fisher Scientific), and cDNA was obtained using a ProtoScript First Strand cDNA Synthesis kit (New England Biolabs). For real-time PCR (RT-qPCR), a FastStart SYBR Green Master kit and LightCycler Nano (Roche) were used. The results were obtained using the 2^−ΔΔCt^ method as a relative quantification strategy for analyzing q-PCR data. Data are presented as relative mRNA levels of the gene of interest normalized to *β*-*ACTIN* mRNA levels (Supplementary Table 1).

### 2.5. Annexin V-FITC/PI

Cell death was evaluated by phosphatidylserine surface expression and detection of the permeability for the cationic marker propidium iodide (PI) using the Annexin V-FITC/PI method. The A549 cell cultures were seeded in six-well plates at 150.000 cells per well. Twelve hours after seeding, the cultures were infected with *sh-RUNX2* or *sh-Ctrl* lentivirus. Twenty-four hours after infection, the cells were treated with the vehicle (dimethyl sulfoxide-DMSO) or 0.2 *μ*m DOXO (apoptosis-inducing agent). After treatment, harvested cells were resuspended in Becton Dickinson Biosciences 1X Annexin Buffer (BD Biosciences, San Jose, CA) and incubated with Annexin V-FITC/PI for 15 minutes at room temperature in the dark. Finally, the results were obtained using a FACSAria II (Becton Dickinson) or CytoFLEX (Beckman Coulter, Waltham, MA, USA) flow cytometer and analyzed with the FlowJo software v10.6.1. This assay was evaluated at time intervals of 24 and 48 hours. Each assay was conducted in duplicate, and three independent experiments were performed.

Annexin V-FITC/PI assays were also performed to evaluate the concentration of DOXO for use in apoptosis assays. This procedure was conducted at time intervals of 24 and 48 hours. The experiments were performed with A549 cell cultures in six-well plates and which the concentrations of 0.05 *μ*m, 0.1 *μ*m, and 0.2 *μ*m of DOXO were assessed (Supplementary Figure 1).

### 2.6. Immunofluorescence Assays

For immunofluorescence assays, cells were grown on sterile coverslips in twelve-well plates. Subsequently, the cells were fixed in 4% paraformaldehyde for 15 minutes and permeabilized with 0.2% Triton X-100 for 15 minutes. After this, they were blocked with BSA (bovine serum albumin) for 30 minutes. A panel of antibodies targeting the BCL2 family (ab228527) was implemented, consisting of one secondary antirabbit antibody (donkey antirabbit IgG H&L Alexa Fluor® 647) and recombinant rabbit monoclonal antibodies against BAD, BAX, BCL2, BCL-XL, and MCL1. The antibody SC-390351 was used to detect RUNX2 (Santa Cruz Biotechnology), and cancer Ep-CAM sc-25308 (Santa Cruz Biotechnology) and HCAM/CD44 sc-7297 (Santa Cruz Biotechnology) were assessed as cancer cell markers. For fluorescence evaluation, 640 × 640-pixel resolution images were obtained with an FV1000 laser scanning confocal microscope (Olympus, Tokyo, Japan) using an UPLSAPO 60 × 1.35 NA oil immersion objective. The images were processed using the free software ImageJ 1.52p (National Institutes of Health, USA). We calculated the corrected total cell fluorescence (CTCF) of each of the proteins evaluated (CTCF = integrated density–area of selected cell × mean fluorescence of background readings) using ImageJ 1.52p software. The required N/C calculation is simply the mean nuclear intensity divided by the mean cytoplasmic intensity and was calculated by subtracting the contribution of background from the integrated fluorescence density within the regions of interest drawn around cells. Columns represent mean values, and error bars represent the standard deviation. An unpaired *t*-test is used to compare means.

### 2.7. ChIP (Chromatin Immunoprecipitation) Assays

Chromatin immunoprecipitation (ChIP) was performed in the lung adenocarcinoma cell line A549. In brief, A549 cells (4 × 10^9^ per ChIP) in 100 mm culture dishes were cross-linked with 1% formaldehyde, and the reaction was quenched by glycine. Cells were then lysed, and nuclei were treated as described in Herreño et al. [10]. Chromatin solutions were incubated with anti-RUNX2 antibody (sc-390351, Santa Cruz Biotechnology). For the ChIP-qPCR experiments, primers were designed against the *MCL*, *BCL-XL*, and *BCL-2* promoters. The primer sequences are listed in Supplementary Table 1 [14].

### 2.8. Statistical Analysis

To determine statistically significant differences, nonparametric tests were employed. A Kolmogorov-Smirnov test was used for comparison of two samples. When more than two groups were compared, a Kruskal-Wallis test was employed followed by Dunnett's multiple comparison tests. For all experiments, three independent biological replicates were assessed. Statistical analyses were performed using GraphPad Prism version 8.0. Data are presented as the median with the 95% CI. A *p* value < 0.05 was considered statistically significant. The significance results of each specific test are indicated in each figure: ^∗^*p* < 0.05; ^∗∗^*p* < 0.01; and ^∗∗∗^*p* < 0.001. For ChIP assays, one-way analysis of variance followed by Dunnett's post hoc test was performed to determine differences.

## 3. Results

### 3.1. *RUNX2* Overexpression in Lung Cancer NSCLC

**Figure 1:**
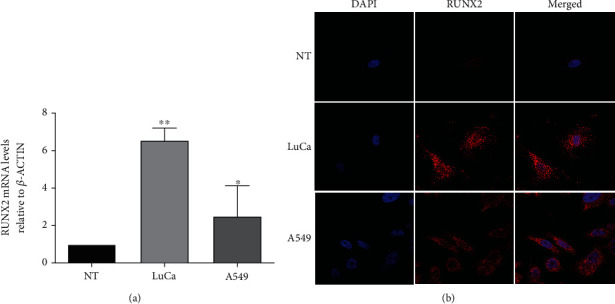
*RUNX2* overexpression in lung cancer. (a) *RUNX2* mRNA levels relative to *β-ACTIN* in lung cancer tissue (LuCa) and lung adenocarcinoma cell line A549 compared with noncancerous tissue (NT). mRNA levels were quantified by RT-qPCR. Statistical analyses were performed with respect to NT (three independent experiments) ^∗^*p* < 0.05, ^∗∗^*p* < 0.01. (b) RUNX2 protein expression by immunofluorescence assays. Images were obtained by confocal microscopy for immunolabelling of the RUNX2 protein (red, Alexa Fluor 546). For the visualization of the nuclei, the fluorescent marker DAPI (blue) was implemented. Images were acquired with the Olympus FV100 confocal microscope with a 60x Plan APO oil objective.

Real-time RT-qPCR was performed to quantify *RUNX2* expression in the lung adenocarcinoma cell line A549 and in tumoral tissue obtained from one lung adenocarcinoma patient (LuCa). We used one sample obtained from nontumor lung tissue (NT) as a control. The clinical characteristics of patients with diagnosis of NSCLC (LuCa) and without a cancer diagnosis (NT) are specified in Supplementary Table 2. *β-ACTIN* was used as an internal control to normalize differences in total RNA levels. The results revealed that tumor tissue (LuCa) and the A549 cell line had higher *RUNX2* expression levels than nontumor tissue (NT) (Figure 1(a)). Protein expression evaluated by immunofluorescence assays showed nuclear and cytoplasmic RUNX2 expression in lung tumor tissue (LuCa) and the A549 cell line (Figure 1(b)).

The quantitative fluorescence intensity analysis for protein level expression of RUNX2 showed higher expression in nucleus than the cytoplasm (Supplementary Figure 1). Likewise, the characterization and identification of CD44 and EpCAM tumor markers evaluated in the A549 cell line, the NT sample, and LuCa are shown in Supplementary Figure 2. Results show positive patterns in tumor cells.

### 3.2. Induction of A549 Cell Death by *sh-RNA*-Mediated Knockdown of RUNX2

To identify the role of the RUNX2 transcription factor in the apoptosis process in lung adenocarcinoma cells, we knocked down RUNX2 using sh-RNA in A549 cells. As shown in Supplementary Figure 3, downregulation of RUNX2 for 48 hours inhibited RUNX2 transcription and translation.

**Figure 2:**
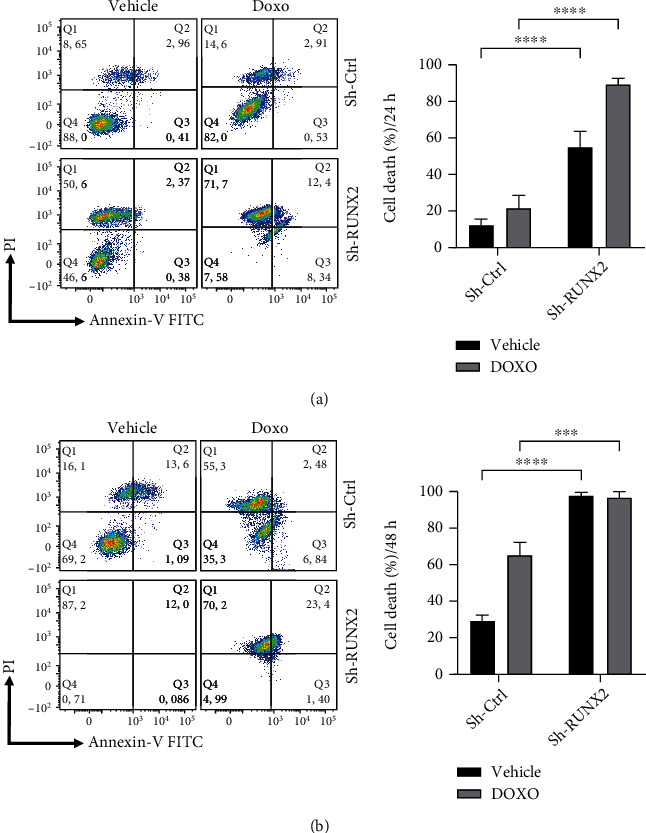
Induction of cell death in A549 lung cancer cells with downregulation of *RUNX2*. A549 cells were treated with doxorubicin or vehicle for 24 h and 48 h after pretreatment with lentiviral particles coding for sh-RNA against *RUNX2*. (a). A representative contour plot of A549 cells treated with DOXO and vehicle for 24 h, pretreated with *sh*-*RUNX2* and *sh*-*Ctrl*, and labeled with Annexin V-FICT and PI is shown. (b). A549 cells were treated with DOXO and vehicle for 48 h, pretreated with *sh*-*RUNX2* and *sh*-*Ctrl*, and labeled with Annexin V-FICT and PI. Right panels show the percentages of dead cells are shown in bars of media ± SEM (three independent experiments) ^∗^*p* < 0.05, ^∗∗^*p* < 0.01, ^∗∗∗^*p* < 0.001.

Subsequently, to determine if RUNX2 is involved with cell death processes, Annexin V-FITC/propidium iodide (PI) assays were implemented in which the externalization of phosphatidylserine (PS) was evaluated as a marker of apoptosis. Cells previously infected with sh-RNA (*sh-RUNX2/sh-Ctrl*) were treated with the vehicle DMSO or apoptosis inducer DOXO. This trial was evaluated at 24 h and 48 h. Previously, a concentration of 0.2 *μ*m DOXO was determined via Annexin V-FITC/PI assays in A549 cells treated at different concentrations (0.05 *μ*m, 0.1 *μ*m, and 0.2 *μ*m). The results of this trial revealed that treatment with DOXO at a concentration of 0.2 *μ*m induced cell death in 11% of the population at 24 hours and 40% at 48 hours (Supplementary Figure 4). The results obtained are shown through Annexin V/PI labeling. Cells in a state of early apoptosis (Annexin V+/PI-), late apoptosis (Annexin V+/PI+), and necrosis (Annexin V-/PI+) were identified (Figure 2). An increase of Annexin V+ cells was observed in A549 cells infected with *sh-RUNX2* compared with cells infected with *sh*-*Ctrl* (Figure 2(a)). Furthermore, in the right panel of Figure 2, the results are shown as a percentage of dead cells, indicating that among both treatment groups (vehicle and DOXO), cells with RUNX2 knockdown (*sh-RUNX2*) presented a higher percentage of cell death at 24 hours. These findings were consistent with what was detected at 48 hours. At 48 hours, highly significant increases in death were observed in cells infected with *sh-RUNX2* (Figure 2(b)). It is essential to highlight that the percentage of cell death induction was near 100% in the two conditions analyzed (vehicle and DOXO).

### 3.3. RUNX2 Absence Was Associated with Decreased Antiapoptotic Proteins in the BCL2 Family

**Figure 3:**
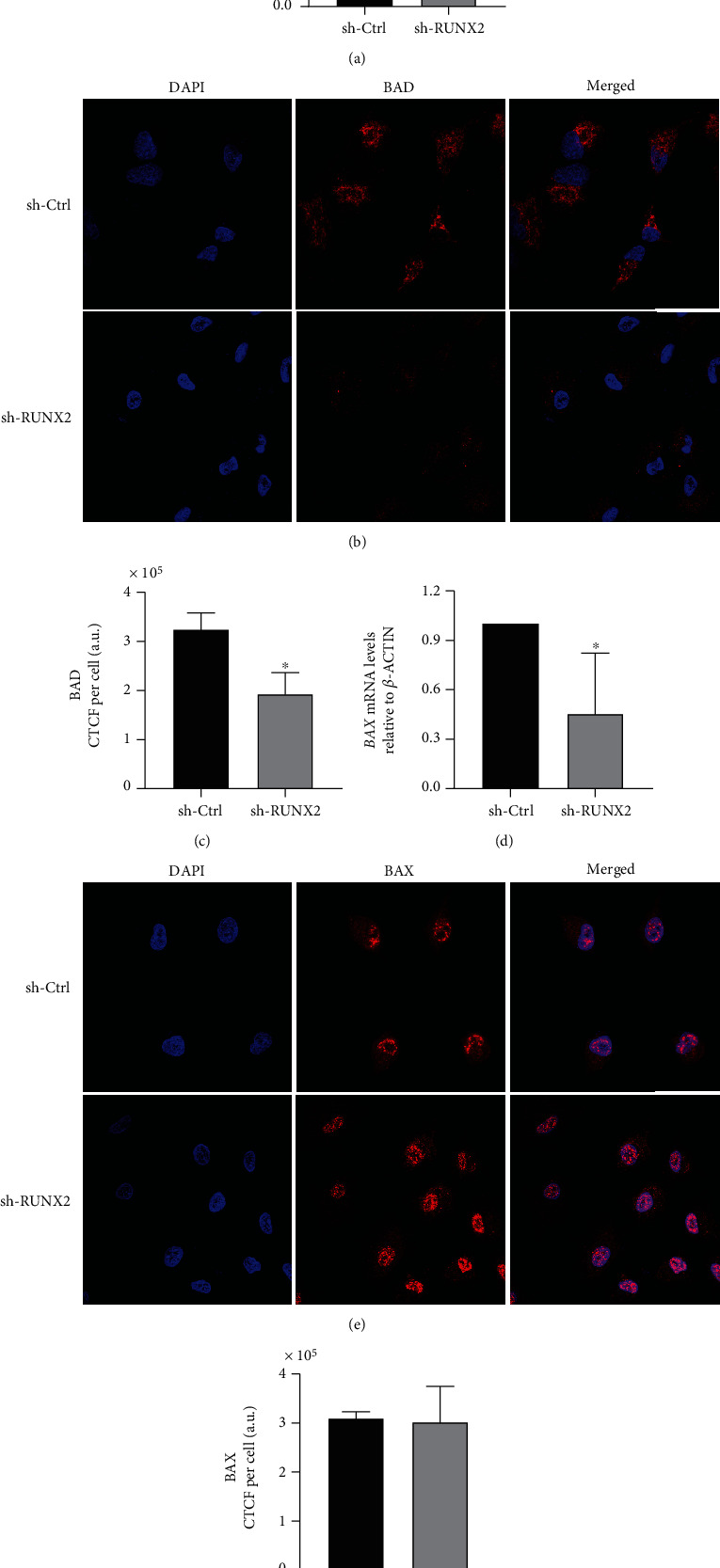
Knockdown of *RUNX2* and proapoptotic gene expression levels: *BAD* and *BAX*. A549 cells were infected with lentiviral particles coding for sh-RNAs against *RUNX2*. BAD (a) and BAX (d) mRNA levels were quantified by RT-qPCR 48 h after infection. Statistical analyses were performed with respect to cells infected with the virus generated with the pLKO.1 empty vector (sh-Ctrl.). ^∗^*p* < 0.05 (three independent experiments). Protein levels were analyzed with immunofluorescence assays. BAD protein expression mitochondrial pattern (b). BAX protein expression with nuclear localization (e). Total cell fluorescence (CTCF) of protein was evaluated (CTCF = integrated density − area of selected cell × mean fluorescence of background readings) using the software ImageJ 1.52p (c, f).

To determine the role of RUNX2 in the apoptosis process, we knocked down *RUNX2* using sh-RNA in A549 cells. Then, we analyzed changes in the expression levels of genes in the *BCL2*-family using RT-qPCR and immunofluorescence assays. For this analysis, gene expression was evaluated in cells infected with lentiviral particles targeting *RUNX2* or containing *sh*-*Ctrl*. A decrease in mRNA of *BAD* and *BAX* (proapoptotic genes) after *sh-RUNX2* treatment was detected (Figures 3(a) and 3(d)). However, the protein levels detected by immunofluorescence assays did not change (Figures 3(b), 3(c), 3(e), and 3(f)).  Figure 3(b) shows that BAD has a mitochondrial protein expression pattern while the BAX exhibits a nuclear expression pattern (Figure 3(e)). Most BAX proteins are found in the cytoplasm. BAX is one of the gatekeepers that control mitochondrial outer membrane permeabilization during the intrinsic apoptosis pathway. However, a strong link between the nuclear localization of the proapoptotic BAX protein and essential cellular functions such as proliferation and migration in lung cellular subtypes has been established [15].

**Figure 4:**
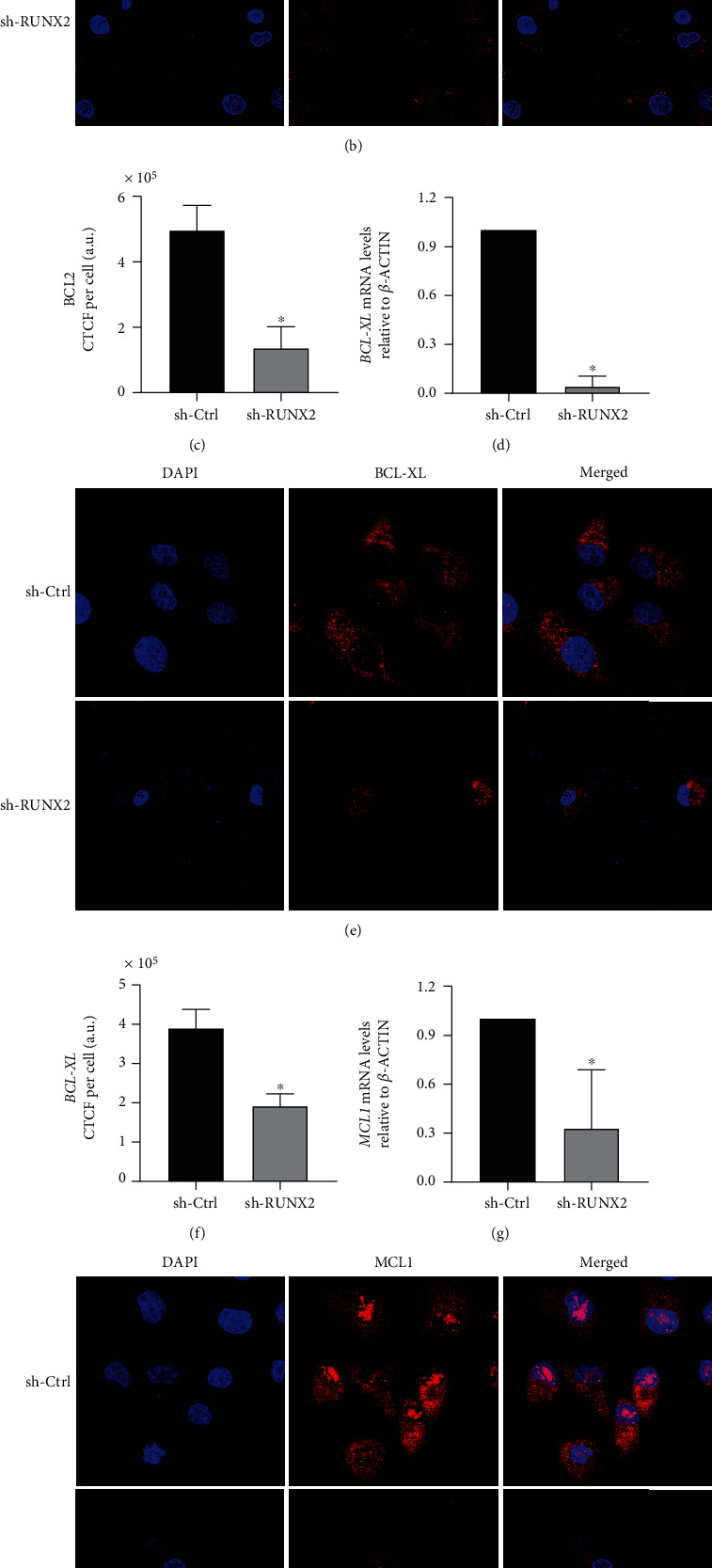
Knockdown of *RUNX2* affects antiapoptotic marker expression BCL2, BCL-XL, and MCL1. A549 cells were infected with lentiviral particles coding for sh-RNAs against *RUNX2*. Antiapoptotic gene expression levels: *BCL2*, *BCL-XL*, and *MCL1*. *BCL2* (a), *BCL-XL* (d), and *MCL1* (g) mRNA levels were quantified by RT-qPCR 48 h after infection. Statistical analyses were performed with respect to cells infected with the virus generated with the pLKO.1 empty vector (sh-Ctrl.). ^∗^*p* < 0.05; ^∗∗^*p* < 0.01 (three independent experiments). Protein levels were analyzed with immunofluorescence assays. BCL-2 protein expression with nuclear localization (b). BCL-XL protein expression with mitochondrial localization (e). MCL1 pattern of mitochondrial protein expression (h). Total cell fluorescence (CTCF) of protein was evaluated (CTCF = integrated density − area of selected cell × mean fluorescence of background readings) using the software ImageJ 1.52p (c, f, i).

In contrast, the analyses of antiapoptotic genes showed statistically significant decreases in the mRNA expression levels of *BCL2*, *BCL-XL*, and *MCL1* (Figures 4(a), 4(d), and 4(g)). These findings were confirmed with immunofluorescence assays, which showed decreases in the protein expression of BCL2 (nuclear expression pattern, Figures 4(b) and 4(c) B and C), BCL-XL (mitochondrial expression pattern, Figures 4(e) and 4(f)), and MCL1 (mitochondrial expression pattern, Figures 4(h) and 4(i)). In summary, our results indicate that the RUNX2 transcription factor participates in the intrinsic pathway of apoptosis avoidance through the transcriptional regulation of antiapoptotic genes *BCL2*, *BCL-XL*, and *MCL1* in lung adenocarcinoma.

**Figure 5:**
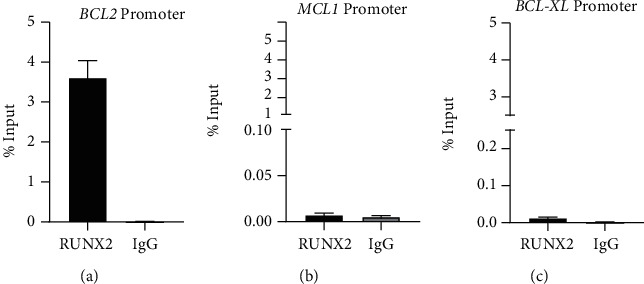
RUNX2 interacts with *BCL2* proximal promoter in lung adenocarcinoma cell line A549. (a) Interaction of RUNX2 with the promoter of *BCL2* gene. ChIP-qPCR assays were performed in lung adenocarcinoma cell line A549. ChIP assays confirm the interaction of RUNX2 protein with promoter region in lung adenocarcinoma. Promoters of *MCL1* (b) and *BCL-XL* (c) were analyzed. Antibody against RUNX2 was used. Results are expressed as % input ± SEM using normal IgG as a specificity control. Results and statistical analyses were performed with respect to normal IgG (specificity control) (three independent experiments).

To confirm whether the regulatory role of the RUNX2 transcription factor on these genes is related to their binding to the promoter region, we performed ChIP assays in A549 cells. As presented in Figure 5(a), RUNX2 was enriched for *BCL2* promoter compared with negative IgG control. In contrast, ChIP assays with *MCL1* and *BCL-XL* did not show enrichment of RUNX2 in their promoter regions (Figures 5(b) and 5(c)).

## 4. Discussion

Master transcription factors are critical developmental regulators that can be used by cancer cells to control the expression of oncogenic transcriptional programs [4]. These proteins are often essential for cancer survival and represent vulnerabilities that can be exploited therapeutically [16]. Runt-related transcription factor (RUNX) proteins belong to a transcription factor family known as master regulators of important embryonic developmental programs [17]. Transcription factor RUNX2 is considered the master regulator of osteoblastic differentiation in humans by regulating multiple signaling pathways and transcriptional activation of a series of downstream molecules [8]. RUNX2 is closely related to the proliferation, invasion, and bone metastasis in multiple cancer types, such as osteosarcoma, breast cancer (BC), prostate cancer, gastric cancer, colorectal cancer, and lung cancer [7–10, 18].

Lung cancer causes the highest number of cancer-related deaths worldwide [19]. Overexpression of *RUNX2* in NSCLC is significantly correlated with tumor size, stage, and lymph node metastasis [8, 10, 20]. Recent studies by our research group have revealed that the overexpression of RUNX2 in lung adenocarcinoma is involved in epithelial-mesenchymal transition through transcriptional regulation of the *VIMENTIN*, *TWIST*1, and *SNAIL*1 genes [10].

In the present study, we detected increases in *RUNX2* expression at the mRNA and protein levels in the A549 lung adenocarcinoma cell line and in tumor tissue of a patient with a diagnosis of lung cancer. To determine RUNX2's role in the apoptosis process in lung adenocarcinoma, loss-of-function assays coupled with Annexin V/PI assays were performed using *RUNX2* sh-RNA in the A549 lung adenocarcinoma cell line. Annexin V/PI assay allows the identification of cells in a state of early apoptosis, late apoptosis, and secondary necrosis through the detection of phosphatidylserine residues, a product of alterations in the apoptotic cell plasma membrane [21]. Furthermore, these assays were performed on cells treated and untreated with the apoptosis-inducing agent doxorubicin. This agent is an inducer of apoptosis that negatively regulates the expression of the antiapoptotic protein BCL2 and positively regulates proteins such as BAX, caspase-8, and caspase-3 [21]. Flow cytometry analyses of cells infected with *sh-Ctrl* and *sh-RUNX2* and treated with the respective treatments (DMSO and DOXO) showed that the silencing of *RUNX2* led to a significant increase in the percentage of cell death in cells treated with DMSO (vehicle), and that this effect was higher when cells were additionally treated with DOXO (Figure 2). This result supports the premise that the transcription factor RUNX2 participates in apoptosis evasion processes in non-small-cell lung cancer. At 48 hours, differences were observed between the percentage of cells in secondary necrosis compared to the results at 24 hours after silencing. Nevertheless, this phenomenon is due to secondary necrosis in which the necrosis process begins after the onset of apoptosis [22].

Our results confirm that apoptosis is a process that is being activated after the loss of function of *RUNX2*. Although RUNX2 has been reported to participate in the apoptotic process in other types of cancer, studies in lung cancer are scarce [7, 23–25]. A role for RUNX2 in apoptosis was first identified by Bellido et al., who showed that the antiapoptotic effect of parathyroid hormone was mediated by this transcription factor in osteoblasts [26]. Similar results have also been found in lymphomas and prostate cancer, where low apoptotic rates have been detected related to overexpression of *RUNX2* and genes such as *Myc* and *BCL2* [23].

To evaluate molecular changes related to the apoptosis process in lung adenocarcinoma, *RUNX2* loss of function was induced using sh-RNA in A549 cells. Our findings demonstrated that cells infected with *sh-RUNX2* entered apoptosis, as shown in Annexin V/PI assay (Figure 2), and the mRNA and protein expression of *BCL2*, *MCL1*, and *BCL-XL* was decreased (Figure 4). On the other hand, proapoptotic proteins BAD and BAX have not shown changes in their protein expression after infection with *sh-RUNX2* (Figure 3). However, it is important to highlight that the results of qPCR assays showed a decrease of their mRNA expression after infection with *sh-RUNX2*. This discordance between mRNA and protein expression levels has been described in different developmental stages or disease conditions [27]. The lack of a significant correlation between mRNA and protein expression could be due to biological reasons. First, complicated biological processes, such as transcriptional splicing, posttranscriptional splicing, translational modifications, translational regulation, and protein complex formation, might affect the relative mRNA and protein levels of various genes to various degrees [27]. Second, different biological or experimental rates of mRNA and protein degradation could affect the mRNA and protein correlations. Third, different mRNA secondary structures can result in different protein translation efficiencies [27]. Furthermore, in certain pathologies, such as lung cancer, these discrepancies have also been observed. In this way, Chen et al. demonstrated that the level of protein abundance in lung adenocarcinomas was associated with the corresponding levels of mRNA in 17% (28 proteins) of 165 proteins examined. This was higher than the amount predicted to result from chance alone (which was 5.1%) and suggests that a transcriptional mechanism likely underlies the abundance of these proteins in lung adenocarcinomas [28].

On the other hand, it is important to note that the results of immunofluorescence assays showed a change of pattern distribution of BAX protein after RUNX2 silencing. In these cells, the results showed an increase in fluorescence signals in the nucleus of cells. Previous reports have shown that BAX proteins are related to mitochondrial membrane permeabilization during apoptosis and that they are localized in the cytoplasm. However, Brayer et al. demonstrated the nuclear localization of BAX protein in lung cellular subtypes, and in this case, their function is related to proliferation and migration [15].

These results indicate that transcription factor RUNX2 is involved in the regulation of *BCL*2, *MCL*1, and *BCL-XL* gene expression. ChIP assays demonstrated that RUNX2-mediated transcriptional regulation of *BCL*2 occurs by direct binding to its promoter region. In contrast, it is likely that the transcriptional regulation of the *MCL1* and *BCL-XL* genes is indirect and requires the presence of other transcription factors or epigenetic enzymes. For example, it has been described that *MCL*1 and *BCL-XL* are transcriptionally modulated in response to different cellular stresses, such as ER stress and hypoxia. In addition, it has been reported that the regulation of the transcriptional levels of *MCL1* and *BCL-XL* is dependent on the binding of HIF1-1*α* to their promoter regions [29, 30]. On the other hand, Kwon et al. reported that the interaction between HIF1-1*α* and RUNX2 is important to activate angiogenic signaling through transcriptional regulation of *VEGF*. In the same study, HIF1 and RUNX2 were shown to physically interact using sites within the RUNX2 Runt domain in mesenchymal cells [31]. In the future, it would be interesting to evaluate this type of interaction in the A549 lung adenocarcinoma cell line.

In prostate cancer, it has been described that RUNX2 can bind to the promoter region of antiapoptotic genes and regulate their expression through the recruitment of other cofactors, such as *BCL*2 [23]. Furthermore, gain-of-function studies of *RUNX*2 in prostate cancer cells identified several pro- and antiapoptotic genes differentially expressed in *RUNX*2-overexpressing cells compared to control cells [23]. The genes described were *BCL*2, *cIAP*2, *STAT5a*, *Casp*14, *TRAIL*, *Casp*5, and *PUMA*, suggesting that RUNX2 may indirectly regulate the expression of various apoptosis-associated genes [23]. As a follow-up to this work, the authors recommend performing RUNX2 gain-of-function studies in our biological model of lung adenocarcinoma to confirm the findings presented above.

## 5. Conclusion

Apoptosis evasion is one of the hallmarks of cancer, and disruption of normal apoptotic regulatory mechanisms in cancer cells can be a significant molecular force driving the progression of the disease [5]. Upregulation of *RUNX2* expression is a feature of many cancer types, including lung cancer, and is associated with proliferation and epithelial-mesenchymal transition. In the present study, we showed the role of RUNX2 in the apoptosis process. Our findings demonstrated that RUNX2 participates in the evasion of apoptosis through direct transcriptional regulation of *BCL2* and indirect regulation of *MCL1* and *BCL-XL* genes.

## Data Availability

The original data shown in Figures 1–4 used to support the findings of this study are available from the corresponding author upon request.

## Supplementary Materials

Supplementary Materials Supplementary Table 1: the list of primers used for RT-qPCR and ChIP assays is detailed. Supplementary Table 2: a description of clinical variables of subjects with nontumor lung tissue (NT) control and NSCLC lung cancer (LuCa). Supplementary Figure 1: shows the fluorescence quantification of RUNX2 protein expression at total cell (CTCF) (A), nuclear CTNF (B), and a nuclear/cytoplasmic ratio (C). Supplementary Figure 2: shows the expression of the cancer cell markers CD44 (A) and EpCAM (B) in LuCa and A549 cells. Supplementary Figure 3: validation data of the RUNX2 knockdown at mRNA level by RT-qPCR (A) and protein by Western blot analyses. Supplementary Figure 4: shows the dispersion diagrams (A) and quantification (B) of Annexin V/PI assays for the determination of optimal DOXO concentration for apoptosis induction in A549 cell line treated with concentrations of 0.05 *µ*m, 0.1 *µ*m, and 0.2 *µ*m of DOXO for 24 h and 48 h.
