# Supplementary material for: Regulatory Role of the RUNX2 Transcription Factor in Lung Cancer Apoptosis
Source: Int J Cell Biol. 2022 Dec 3;2022:5198203. doi: 10.1155/2022/5198203 (PMC9741537; doi:10.1155/2022/5198203)
Supplement: Supplementary Materials — Supplementary Table 1: the list of primers used for RT-qPCR and ChIP assays is detailed. Supplementary Table 2: a description of clinical variables of subjects with nontumor lung tissue (NT) control and NSCLC lung cancer (LuCa). Supplementary Figure 1: shows the fluorescence quantification of RUNX2 protein expression at total cell (CTCF) (A), nuclear CTNF (B), and a nuclear/cytoplasmic ratio (C). Supplementary Figure 2: shows the expression of the cancer cell markers CD44 (A) and EpCAM (B) in LuCa and A549 cells. Supplementary Figure 3: validation data of the RUNX2 knockdown at mRNA level by RT-qPCR (A) and protein by Western blot analyses. Supplementary Figure 4: shows the dispersion diagrams (A) and quantification (B) of Annexin V/PI assays for the determination of optimal DOXO concentration for apoptosis induction in A549 cell line treated with concentrations of 0.05 µm, 0.1 µm, and 0.2 µm of DOXO for 24 h and 48 h. [file 5198203.f1.zip › 5198203.f1/Supplementary Tables.docx]

# Supplementary Table 1. Primer sequence of RT-qPCR and ChIP assays.



**PRIMER SEQUENCE**


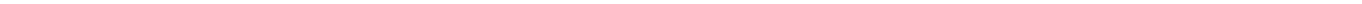
**Target Gen Forward Reverse**

| **mRNA expression qPCR primers** | |  |
| --- | --- | --- |
| RUNX2 | 5'-GTCACTGTGCTGAAGAGGCT-3' | 5'-GGTTAATCTCCGCAGGTCAC-3' |
| BAD | 5´-GAGTGAGCAGGAAGACTCCAGC-3 | 5'-TCCACAAACTCGTCACTCATCC-3' |
| BAX | 5´-CCCGAGAGGTCTTTTTCCGAG-3' | 5´-CCAGCCCATGATGGTTCTGAT-3' |
| BCL XL | 5´-TCCTTGTCTACGCTTTCCACG-3' | 5'-GGTCGCATTGTGGCCTTT-3' |
| MCL1 | 5'-CCAAGGCATGCTTCGGAAA-3' | 5'-TCACAATCCTGCCCCAGTTT-3' |
| BCL2 | 5’- GCCTTCAAGGTGGTAGCCC-3’ | 5’- CGTTACCCGCCATGACAGA-3’ |
| B-ACTINA | 5´-AGAGCTACGAGCTGCCTGAC-3' | 5'- AGCACTGTGTTGGCGTACAG-3' |
| **ChIP qPCR primers** | | |
| MCL1 | 5’-TAGGTGCCGTGCGCAACCCT-3’ | 5’-ACTGGAAGGAAGCGGAAGTGAGAA-3’ |
| BCL-XL | 5’-AGGGTAAATGGCATGCATATTAA-3’ | 5’-TTATAATAGGGATGGGCTCAACCA-3’ |
| BCL2 | 5’-CCCTATTAAGTAAGCCGCTGTG-3’ | 5’-GTACGCGCAAGCAGACAGT-3’ |

#
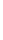
Supplementary Table 2: Description of clinical variables of subjects with non-tumor lung tissue (NT) control and NSCLC lung cancer (LuCa).

| **Clinical characteristics** | **Non tumoral (NT)** | **NSCLC case (LuCa)** |
| --- | --- | --- |
| Sex | Male | Female |
| Age | 83 years | 67 years |
| Histological tumor type | -- | Squamous cell carcinoma |
| Lung cancer origin | -- | Primary |
| TNM stage | -- | IIIB |
| Immunohistochemical markers | -- | P63 |
| Comorbidity | None | Breast and uterine cancer |
| Exposure history  (Wood or cigarette smoke) | None | None |
